# Gastric cancer organoids: a promising model for personalized treatment of gastric cancer

**DOI:** 10.3389/fcell.2025.1712621

**Published:** 2026-01-29

**Authors:** Yu Chang, Yu Liu, Huahua Zhang, Xia Lei, Yunfeng Hu

**Affiliations:** 1 Department of Radiation Oncology, The First Affiliated Hospital of Yan’an University, Yan’an, Shaanxi, China; 2 Medical Research and Experimental Center, Yan’an Medical College, Yan’an University, Yan’an, Shaanxi, China; 3 Department of Gynecology, The First Affiliated Hospital of Yan’an University, Yan’an, Shaanxi, China

**Keywords:** gastric cancer, organoid model, individualized treatment, translational medicine, challenges and prospects

## Abstract

Gastric cancer is one of the most prevalent malignant tumors in the gastrointestinal tract. At present, the main treatment methods of gastric cancer include chemotherapy, targeted therapy and immunotherapy. However, due to the strong heterogeneity of tumor cells, the current treatment schemes have different effects on each patient. Therefore, individualized treatment strategies for different patients have become an inevitable trend. The organoid model is an emerging technology for three-dimensional (3D) cell culture *in vitro*, which can simulate the three-dimensional structure of real organs and the interaction between cells, while retaining the heterogeneity of patients’ tumor cells. The response of organoid model to treatment can replace the response of patients’ tumor cells to treatment. Organoid model of gastric cancer can effectively bridge the basic research and clinical practice, and show remarkable transformation potential and broad application prospects in promoting individualized treatment of gastric cancer. In this paper, the establishment methods of organoid model of gastric cancer are systematically reviewed, and its application in drug sensitivity detection, biomarker discovery and mechanism research is discussed, in order to provide theoretical basis and practical reference for the development of precise treatment strategy for gastric cancer.

## 1 Introduction

Gastric cancer is one of the most common malignant tumors of the digestive tract, with nearly one million new cases diagnosed annually and over 650,000 deaths worldwide each year (Sundar et al., 2025). Surgery, chemotherapy, radiotherapy, immunotherapy, and targeted therapy are the main treatment modalities for gastric cancer, and the staging and classification of gastric cancer play a crucial guiding role in determining the optimal treatment strategy (Joshi and Badgwell, 2021; Hirata et al., 2023). Currently, the staging of gastric cancer primarily follows the TNM system, which is based on the depth of tumor invasion, lymph node metastasis status, and presence of distant metastasis (Chen et al., 2021). Early gastric cancer (EGC) refers to tumors confined to the mucosal or submucosal layer, regardless of lymph node metastasis. For patients with EGC, treatment options include endoscopic mucosal resection (EMR), endoscopic submucosal dissection (ESD), or surgical resection (Conti et al., 2023; Espinel et al., 2015). The American Society for Gastrointestinal Endoscopy (ASGE) has put forward precise recommendations for selecting endoscopic or surgical treatment for EGC, based on lesion size, differentiation grade, and ulceration status: For non-ulcerated EGC with well-differentiated or moderately differentiated pathological features, if the lesion size ranges from 20 to 30 mm, ESD is recommended as the preferred treatment. If the lesion size is < 20 mm, either ESD or EMR can be chosen according to clinical practice. However, for poorly differentiated EGC, surgical resection is recommended regardless of lesion size to ensure therapeutic efficacy (Al-Haddad et al., 2023). For locally advanced gastric cancer (LAGC), the main treatment strategy currently centers on surgery combined with perioperative therapy (Sugawara et al., 2021). The 2025 National Comprehensive Cancer Network Clinical Practice Guidelines in Oncology (NCCN) Guidelines classify perioperative chemotherapy as a Category I recommendation for patients with resectable T2 or higher-stage tumors. For certain patients with microsatellite instability-high (MSI-H)/deficient mismatch repair (dMMR) tumors, neoadjuvant therapy or perioperative immunotherapy may be considered (Ajani et al., 2025). Preoperative neoadjuvant therapy can reduce tumor size, downstage the tumor, and thereby improve the surgical resection rate. Postoperative adjuvant therapy, on the other hand, can eliminate minimal residual lesions left after surgery and reduce the risk of recurrence. Systemic therapy is the primary treatment for patients with advanced or metastatic gastric cancer. It aims to control tumor progression, alleviate patient symptoms, and prolong survival. The sixth edition of the Japanese Gastric Cancer Treatment Guidelines states that chemotherapy can reduce tumor size in patients with unresectable advanced or recurrent gastric cancer, but it is difficult to achieve a cure with chemotherapy alone. HER2 testing is strongly recommended for patients with unresectable advanced or recurrent gastric cancer. For HER2-negative patients, first-line treatment is based on chemotherapy, with commonly used regimens including FOLFOX, XELOX, and SOX. For HER2-positive patients, combination therapy with trastuzumab plus chemotherapy is recommended. For advanced gastric cancer, second-line and subsequent-line treatments (therapies administered when first-line treatment fails, disease progresses, or patients cannot tolerate the side effects of first-line therapy) require selecting an appropriate treatment plan based on the patient’s pathological type, general condition, and disease progression status. Targeted agents, immunotherapy, or best supportive care are the main treatment options for patients with advanced gastric cancer, with core goals of controlling tumor progression, alleviating clinical symptoms, and ultimately improving patients’ quality of life (Japanese Gastric Cancer Association, 2023). Patients with early gastric cancer have a favorable prognosis. However, due to the lack of obvious early symptoms, gastric cancer is difficult to detect in its early stages, and most patients are diagnosed at an advanced stage, resulting in a poor prognosis (Jin et al., 2022).

Although a diversified treatment system for gastric cancer—encompassing surgery, chemotherapy, targeted therapy, and immunotherapy—has been established, some patients still achieve suboptimal therapeutic outcomes. This is mainly attributed to the presence of tumor heterogeneity and drug resistance (Kuwata, 2024; Liu et al., 2024). Tumor heterogeneity refers to the presence of cell populations with distinct genetic profiles during tumor cell growth, and these different cell populations may give rise to diverse phenotypes (Xu et al., 2024a). The existence of such intrinsic heterogeneity results in significant variations in the response of different subpopulations to the same treatment regimen within the tumor lesions of a single patient: some subpopulations may be effectively inhibited, while others remain unaffected, ultimately compromising the overall treatment effect. Meanwhile, the development of drug resistance poses a major challenge in current gastric cancer treatment. On one hand, some patients exhibit primary resistance to chemotherapeutic agents due to inherent drug resistance-related genes in their tumor cells, leading to no response from the onset of treatment (Chern et al., 2020). On the other hand, even if the initial treatment is effective, tumor cells may gradually develop acquired drug resistance during therapy through mechanisms such as drug resistance-related gene mutations (Lim and Ma, 2019; Du et al., 2025; Marín et al., 2023), signal pathway reprogramming (BeLow and Osipo, 2020), or alterations in the tumor microenvironment (Mishra et al., 2021), resulting in disease progression in the advanced stage of treatment. In summary, the existence of tumor heterogeneity and drug resistance represents a critical bottleneck in the current management of gastric cancer.

In recent years, personalized precision therapy has become a key focus in the field of oncology research. By addressing the heterogeneity of each patient’s tumor, it enables the selection of tailored treatment regimens, thereby overcoming the limitations of the traditional “one-size-fits-all” approach. Personalized therapy offers significant advantages in improving the accuracy of cancer treatment, reducing adverse effects, and enhancing therapeutic outcomes (Singh et al., 2024). Traditional oncology research has largely relied on cell line models. However, during long-term *in vitro* passaging, these models gradually lose the genomic diversity, cellular subpopulation composition, and microenvironmental interactions characteristic of primary tumors, ultimately diminishing tumor heterogeneity. As a result, their response to treatment often deviates significantly from clinical observations in patients, making them unreliable predictors of real-world therapeutic efficacy (Habanjar et al., 2021). In contrast, tumor organoid models, derived directly from patient primary tumor cells and cultured using three-dimensional (3D) techniques, can recapitulate the *in vivo* 3D architecture of tumor growth while preserving key components of the tumor microenvironment. Moreover, they retain the intrinsic heterogeneity of each patient’s tumor (Zeng et al., 2024). These patient-derived organoid models exhibit treatment responses that closely mirror the clinical outcomes of individual patients, serving as highly accurate *in vitro* surrogates for assessing therapeutic sensitivity (Kretzschmar, 2021). Therefore, gastric cancer organoid models not only hold great promise as tools for screening treatment sensitivity and guiding clinical decision-making but also demonstrate unique value in studying the mechanisms of gastric cancer progression. They serve as a critical bridge connecting basic research and clinical translation.

**FIGURE 1:**
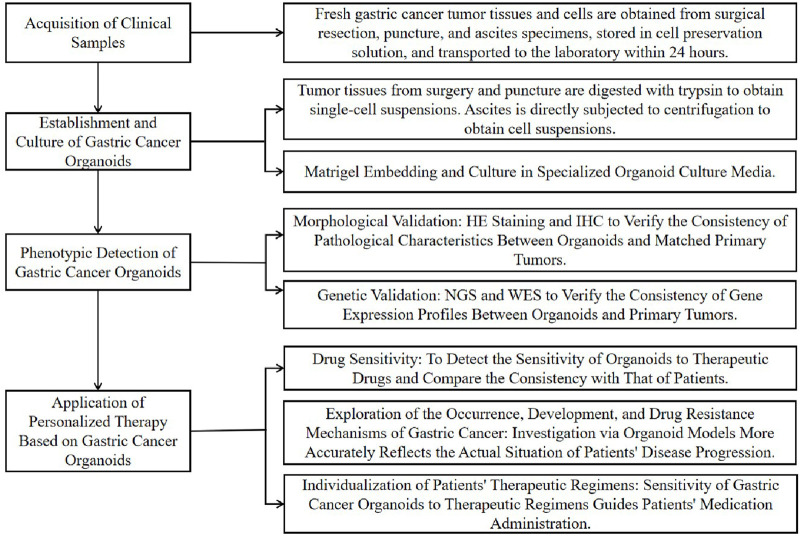
Flow chart of organoid application in personalized cancer therapy for gastric cancer.

In this review, we systematically summarize the key research advances in gastric cancer organoid models. On one hand, we focus on their establishment techniques and phenotypic validation methods. On the other hand, we provide an in-depth analysis of their applications in personalized gastric cancer therapy. Furthermore, we identify the critical bottlenecks hindering the translation of gastric cancer organoids from basic research to clinical practice and discuss their future prospects in precision medicine and drug development. This review aims to serve as a reference for advancing the clinical translation of this model. In this review, we systematically summarize the establishment and application of gastric cancer organoid models (Figure 1). On the one hand, we focus on their technical system for establishment and phenotypic validation methods; on the other hand, we conduct an in-depth analysis of the application scenarios of this model in individualized treatment for gastric cancer.

## 2 Establishment and phenotypic validation of gastric cancer organoid models

Organoid models are one of the most widely concerned research models in the field of current clinical medicine. It mainly refers to mini-organs derived from primary tissues or stem cells under *in vitro* 3D cell culture conditions, which possess self-renewal capacity and are highly similar to *in vivo* tissues in terms of cellular composition and function. Organoids closely resemble *in vivo* primary tissues in genomic expression patterns, cellular heterogeneity, and physiological functions, providing an ideal research tool for recapitulating the physiological and pathological characteristics of organs *in vitro* (Huo et al., 2023; Broda et al., 2019). Based on these properties, the successful establishment of gastric cancer organoid models holds irreplaceable clinical value in the translation into personalized treatment for gastric cancer. This section will focus on the establishment of gastric cancer organoid models, the establishment of biobanks, and phenotypic validation.

### 2.1 Cultivation of gastric cancer organoids and establishment of biological sample banks

**FIGURE 2:**
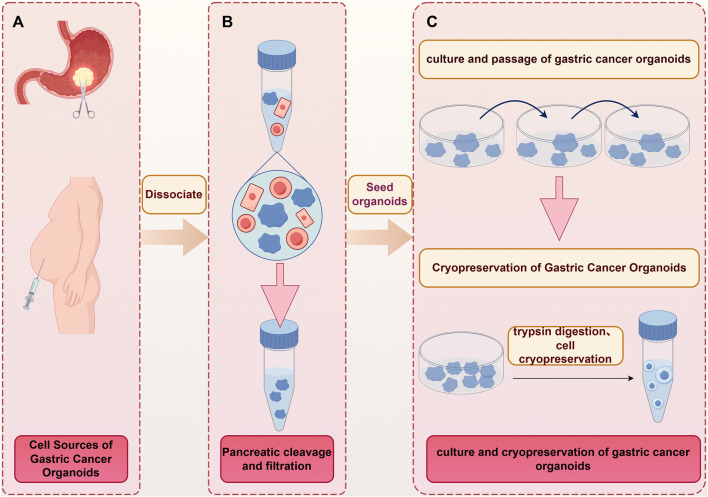
Establishment process of the gastric cancer organoid. **(A)** Acquisition of gastric cancer organoid specimens: Gastric cancer organoid specimens are mainly derived from ascites or gastric cancer surgical specimens; **(B)** Trypsin digestion: Trypsin digests the tissue into single cells, removes other types of cells, and leaves the cells to be further cultured; **(C)** Embedding culture of organoids: Mix the cells with Matrigel evenly, seed them in cell culture plates for culture, passage and cryopreservation. This figure was drawn with Figdraw.

The samples for constructing gastric cancer organoids can be obtained from surgically resected tissues or endoscopic biopsies of primary gastric cancer, or by isolating tumor cells from ascites samples of advanced gastric cancer patients, followed by organoid culture (Huo et al., 2023; Broda et al., 2019; Song et al., 2022). The establishment of gastric cancer organoids is a multi-step systematic process, primarily including sample collection, trypsin digestion, organoid culture, and cryopreservation (Figure 2). Among these, the time from sample collection to processing, the number of tumor cells, and cell viability are critical factors determining the success of establishment (Li H. et al., 2022). Regarding the timeliness of sample processing, for tissue samples obtained from surgical resection or endoscopic biopsy, they must first be minced into small fragments and transferred into specialized cell preservation solution within 30 min after collection, ensuring the tissue is fully immersed to maximize cell viability (Shimono et al., 2019). The preserved tissue specimens should then be immediately transported to the laboratory for aseptic processing in a biosafety cabinet. First, the tissue samples are repeatedly washed with ice-cold PBS buffer containing antibiotics to remove impurities and prevent microbial contamination. Subsequently, forceps and scissors are used to remove adipose tissue and necrotic areas to avoid interference with subsequent culture (Zou et al., 2022). After tissue preprocessing, the samples are minced into fragments smaller than 1 mm in diameter, followed by digestion with an appropriate concentration of tissue dissociation enzyme. Once the tissue is completely dissociated into single cells, a cell strainer is used for filtration to obtain a single-cell suspension (Zhang et al., 2021). The filtered single-cell suspension is then mixed with extracellular matrix (Matrigel) at an optimal ratio. Gentle pipetting is required during mixing to prevent excessive bubble formation, which may impair cell attachment. The mixture is evenly seeded into a 24-well cell culture plate and immediately placed in a 37 °C, 5% CO_2_ incubator for 30 min to facilitate matrix solidification and the formation of a three-dimensional culture scaffold. After complete matrix solidification, pre-prepared organoid complete medium is added to each well, and the plate is returned to the same culture conditions for continuous incubation (Shimono et al., 2019). It is important to note that gastric cancer organoid culture medium differs fundamentally from traditional cell line media. Organoid medium is serum-free and based on a basal medium (Advanced DMEM/F12), supplemented with key growth factors such as epidermal growth factor (EGF) and fibroblast growth factor (FGF). The ratio of growth factors can be dynamically adjusted based on the actual growth status of the organoids (Shimono et al., 2019). During organoid culture, daily observation under an inverted microscope is necessary to monitor growth status (including size, morphology, transparency, and signs of apoptosis). Once the organoids reach sufficient quantity and size, they can be passaged or cryopreserved for long-term stable storage. Currently, the establishment of organoids for malignant tumors such as gastric cancer, colorectal cancer, endometrial cancer, and lung cancer has achieved considerable maturity, with a success rate of over 60%. In contrast, the success rate for organoid establishment of other malignant tumors including prostate cancer and gallbladder cancer is less than 50% (Jiang et al., 2024; Thorel et al., 2024).

Despite significant advancements in the prevention, diagnosis, and treatment of gastric cancer, its incidence and mortality rates remain persistently high. The complexity and heterogeneity of tumor tissues constitute a major barrier to antitumor therapy, and how to mimic the *in vivo* survival environment of tumor tissues *in vitro* while preserving their heterogeneity has become a key focus of current research. Gastric cancer organoids not only can well preserve the genetic and functional characteristics of the original tumor tissues but also can highly recapitulate their *in vivo* survival microenvironment. Therefore, the establishment of a gastric cancer organoid biobank will provide a reliable source of living biological specimens for both basic and clinical research (Li H. et al., 2022; Yan et al., 2018).

Organoid biobanks hold irreplaceable value in disease research, offering living biological samples that closely mirror the phenotypic and molecular profiles of parental tissues while precisely capturing disease heterogeneity. These biobanks serve as critical tools for investigating disease mechanisms and developing personalized treatment strategies. In oncology research, multiple high-impact studies have demonstrated the high concordance between organoids and clinical samples, as well as their translational potential. Ganesh et al. established an organoid biobank comprising 65 colorectal cancer patients, with sample sources covering primary, metastatic, and recurrent tumors. The results clearly confirmed that organoids are not only highly consistent with patients’ original tumor tissues in phenotype but also can accurately reflect patients’ clinical responses to treatment. Meanwhile, the team constructed patient-derived xenograft (PDX) models that recapitulate the initiation, invasion, and migration capabilities of rectal malignant tumors. This successfully established model provides a reliable *in vitro* tool for screening personalized treatment regimens for patients at different stages (Ganesh et al., 2019). Similarly, Yan et al. established a living biobank consisting of 17 normal tissue organoids and 46 gastric cancer organoids. Whole-exome sequencing results showed that the gene expression profiles of *in vitro* cultured gastric cancer organoids are highly consistent with those of *in vivo* cancer cells. More importantly, organoids can maintain excellent genetic stability during long-term culture, with rare additional gene mutations, ensuring the reliability and reproducibility of research results. The team further performed drug sensitivity testing using the successfully constructed gastric cancer organoid models and found that the organoids’ responses to drugs are highly consistent with the post-treatment responses of clinical patients, further demonstrating the potential of organoids in tumor precision therapy (Yan et al., 2018). For rare diseases, due to their extremely low incidence rates, clinical research often faces scarcity of samples and thus becomes difficult to conduct. Organoid biobanks, through *in vitro* establishment and long-term preservation, can provide continuously available living biological samples for rare disease research, break through the bottleneck of insufficient sample size, and supply a large number of biological specimens to accelerate the progress of rare disease treatment (Kondo, 2021).

### 2.2 Phenotypic profiling and validation of gastric cancer organoids

**FIGURE 3:**
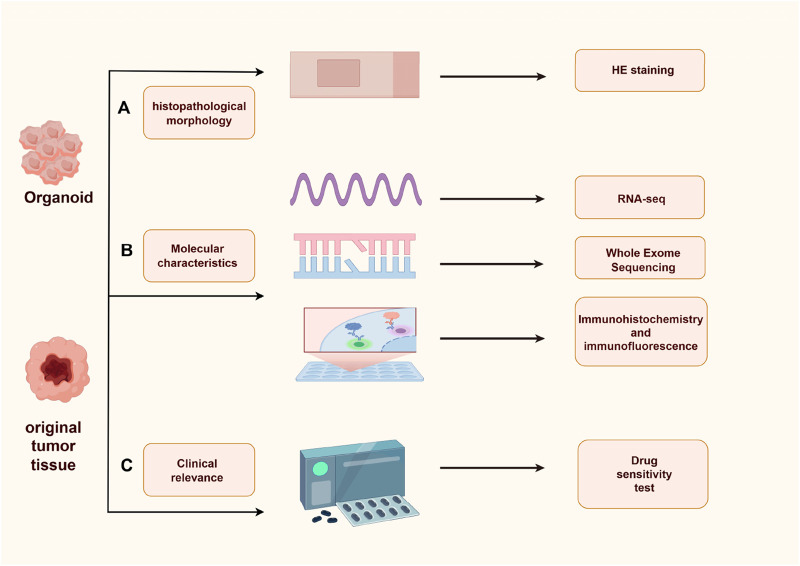
Verification method of gastric organ phenotype. **(A)** HE staining: Consistency of histopathological characteristics between gastric cancer organoids and primary tissue; **(B)** RNA-seq, WES, immunohistochemistry and immunofluorescence: Consistency in gene expression, mutations, and protein expression; **(C)** Drug sensitivity assay: Consistency of drug response between organoids and primary tissue. This figure was drawn with Figdraw.

Gastric cancer organoids are a key tool for simulating gastric cancer progression and tumor cell survival characteristics *in vitro*, and their phenotypic validation is crucial for ensuring model reliability and clinical translational value. However, whether this model can fully replicate all characteristics of patient tissues *in vivo* still requires further research and validation (Wood and Ewald, 2021). Currently, phenotypic validation of gastric cancer organoids primarily focuses on four dimensions: morphology, molecular features, functional characteristics, and clinical relevance (Wallaschek et al., 2019) (Figure 3).

The core of morphological validation lies in observing the growth patterns and cellular morphology of organoids under a microscope, and combining HE staining technology to compare organoid sections with sections of the patient’s own gastric cancer tissue. The consistency is mainly evaluated by examining key features such as the structural characteristics of gastric glands, nuclear-cytoplasmic ratio, and tumor subtype characteristics, thereby determining whether the organoids are consistent with the histopathological characteristics of the original tumor (Lee et al., 2018). Validation of molecular characteristics is the key to confirming the homology between gastric cancer organoids and the original tumor, which is mainly carried out from three aspects: molecular markers, gene expression profiles, and genetic mutation characteristics. Immunohistochemistry and immunofluorescence techniques can be used to detect the expression of gastric cancer-specific molecular markers, such as carcinoembryonic antigen (CEA), carbohydrate antigen 19-9 (CA19-9), carbohydrate antigen 72-4 (CA72-4), alpha-fetoprotein (AFP), and carbohydrate antigen 125 (CA125) (Matsuoka and Yashiro, 2018). Transcriptome sequencing can be used to analyze the correlation of gene expression profiles between organoids and the original tissue, while whole-exome sequencing can compare the mutation characteristics of the two. These two techniques are core methods for verifying the molecular homology between gastric cancer organoids and primary tumors. Existing studies have confirmed that gastric cancer organoids not only have a high degree of consistency with the gene expression profile of primary tumors, but also have a significant correlation in mutation characteristics with the latter. Moreover, organoids do not exhibit high-frequency mutations during passaging, which provides key evidence for their potential to replace original tissue samples at the genetic level. For example, Zhao et al. constructed 57 organoid models derived from gastric cancer patients. Through RNA sequencing, they systematically analyzed the gene expression patterns of organoids and their corresponding primary tumor tissues. The results showed that the gene expression characteristics of the two were highly similar, which further confirmed that gastric cancer organoids can faithfully reproduce the characteristics of primary tumors at the gene expression level and have the potential to replace original tissues for related research (Zhao et al., 2024). Li et al. extracted corresponding tumor cells from the ascites of gastric cancer patients and constructed 11 organoid models. Through immunohistochemistry and whole-exome sequencing, they compared the histological structure and gene expression profile of organoids with those of original ascites tumor cells in histopathology, and found that the two were highly consistent (Li et al., 2019).

Clinical relevance validation is a core step for the translation of organoid models into clinical practice. It mainly involves systematically analyzing the response characteristics of organoid models to treatment *in vitro* and correlating them with the *in vivo* therapeutic efficacy of patients who receive the same treatment, so as to evaluate the clinical predictive ability of organoid models and provide strong evidence for their clinical application. A study on biliary tract cancer successfully established an organoid biobank from 61 biliary tract cancer patients among 82 tumors (success rate: 74.4%). Drug screening experiments were performed on the successfully constructed organoids, and 13 biliary tract cancer patients who received chemotherapy and underwent drug screening in their derived organoids were followed up for 4–26 months. The results showed that three patients were sensitive to the clinical treatment regimen, consistent with the drug response of their derived organoids; one patient was sensitive to 5-FU, but their organoid showed a moderate response to 5-FU. Three patients were resistant to chemotherapy, among whom two had consistent drug responses with their organoids. In addition, four out of six advanced biliary tract cancer patients achieved partial response or disease stability. Organoid drug screening indicated that two patients sensitive to chemotherapy experienced disease progression after adjuvant chemotherapy, among whom one organoid showed significant resistance to chemotherapy and the other showed a moderate response. The results of this study demonstrated that organoids are a useful tool for drug screening and drug response prediction in biliary tract cancer patients (Ren et al., 2023). Zhao et al. successfully established 57 patient-derived gastric cancer organoids from 73 gastric cancer patients (success rate: 78%). First, through HE staining, immunohistochemistry and other tests, it was confirmed that these organoids were highly consistent with the patients’ original tumor tissues in terms of gene expression profiles and histopathological characteristics. Furthermore, the responses of 41 gastric cancer organoids to six commonly used chemotherapeutic drugs for gastric cancer were detected, and correlation analysis was conducted with the patients’ clinical treatment responses. The results confirmed that organoids derived from patients sensitive to chemotherapeutic drugs corresponded to patients with better therapeutic effects in clinical treatment (Zhao et al., 2024).

In summary, phenotypic validation of organoid models is a multidimensional and systematic process requiring comprehensive assessment through morphological, molecular, and clinical correlation analyses to ensure accurate recapitulation of the core phenotypic characteristics of *in vivo* gastric cancer tissues. The high phenotypic concordance between gastric cancer organoids and primary tumor tissues not only validates the reliability and stability of these models but also establishes a robust foundation for their applications in investigating gastric cancer pathogenesis, high-throughput drug screening, personalized therapy optimization, and prognostic biomarker discovery. This advancement significantly accelerates the translation of basic research findings into clinical practice for gastric cancer management.

## 3 Prospects of gastric cancer organoids in personalized therapy

**FIGURE 4:**
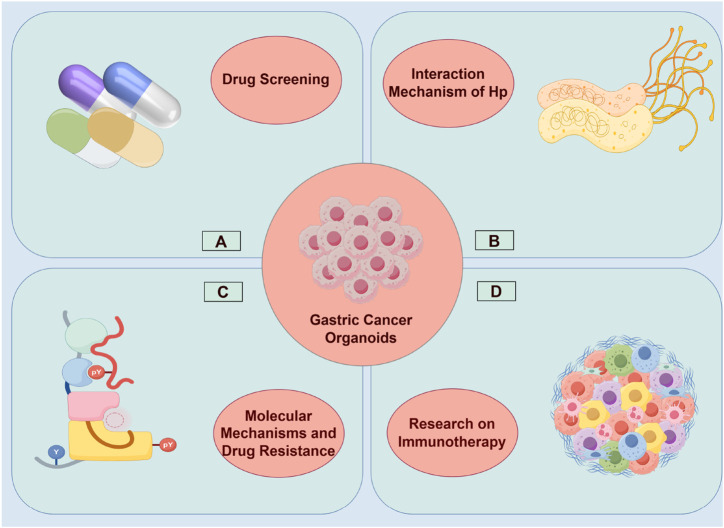
Application of Gastric Cancer Organoids in Personalized Therapy. This figure was drawn using Figdraw. **(A)** Gastric organoids for drug screening; **(B)** Gastric organoids for investigating the carcinogenic mechanism of *Helicobacter pylori*; **(C)** Gastric organoids for immunotherapy; **(D)** Gastric organoids for exploring the molecular mechanisms of gastric cancer progression and chemoresistance.

As *in vitro* representations of patient tumors, tumor organoid models play a pivotal role in guiding personalized treatment strategies. Current research demonstrates that organoids hold significant potential in multiple areas, including drug screening, discovery of novel diagnostic biomarkers, investigation of drug mechanisms and resistance, exploration of combination therapies, and studies on rare diseases and immunotherapy (Xiang et al., 2024; Miao et al., 2022). This section will focus on the potential clinical applications of gastric cancer organoids in therapeutic decision-making (Figure 4).

### 3.1 Predictive value of gastric cancer organoids in treatment sensitivity assessment

As previously mentioned, gastric cancer exhibits significant intertumoral heterogeneity, which is the primary reason for the substantial variability observed in patient treatment responses. Organoid models uniquely preserve this tumor heterogeneity for each patient, with their therapeutic responses accurately reflecting individual patient drug sensitivity. Xu et al. systematically evaluated the sensitivity of 21 gastric cancer organoid lines and 18 normal gastric organoids to five conventional chemotherapeutic agents and 22 targeted therapies. Their findings demonstrated marked variability in drug responses across different organoid lines, closely mirroring the clinical heterogeneity of gastric cancer. This study confirmed that gastric cancer organoids can reliably assess both drug sensitivity and resistance, thereby providing critical guidance for selecting personalized treatment regimens (Xu et al., 2024b).

The research team led by Xiao also provided new clues for the screening of gastric cancer therapeutic drugs through their study: they constructed three patient-derived gastric cancer organoids, and after verifying their consistency with the original tumor tissues, conducted drug sensitivity tests for 5-FU, epirubicin, and nab-paclitaxel based on these organoids. The results showed that each patient-derived organoid exhibited a unique response to 5-FU, epirubicin, and nab-paclitaxel. This characteristic precisely reflects tumor heterogeneity and confirms that organoids retain this key feature of tumors (Xiao et al., 2020). Zu and colleagues successfully constructed 12 gastric cancer organoids from 13 patients (success rate: 92.3%) and detected their sensitivity to 17 chemotherapeutic drugs (including oxaliplatin, 5-FU, hydroxycamptothecin, paclitaxel, docetaxel, carboplatin, vincristine, doxorubicin, and mitomycin), three targeted drugs (entrectinib, larotrectinib, and DS-8201), and one monoclonal antibody drug (trastuzumab). The results showed that even with the same pathological type, tumor stage, and molecular characteristics, organoids displayed different drug responses. Further comparison of the organoid drug screening results with the clinical responses of matched patients indicated that the drug sensitivity test results of organoids were consistent with the clinical responses of six out of eight patients (Zu et al., 2023). Li et al. used 3D organoid culture technology to successfully construct 12 gastric cancer organoids from 26 gastric cancer tissue samples. A series of verifications confirmed that these organoids retain the histological and genetic characteristics of cancerous tissues. Drug sensitivity tests revealed that organoids from different patients exhibited distinct drug responses, fully demonstrating tumor heterogeneity (Li G. et al., 2022). Yoon and team members successfully constructed 13 organoids from 24 patients with locally advanced gastric adenocarcinoma (success rate: 54%). Verification showed that organoids derived from gastric cancer patients were consistent with their original tumor tissues in terms of gene expression profiles, histopathological characteristics, and therapeutic drug responsiveness, and could be used to predict resistance to neoadjuvant chemotherapy in gastric cancer patients (Yoon et al., 2023). From the current relevant research results, it can be seen that gastric cancer organoid models have important value in predicting the efficacy of anti-cancer drugs, providing a new model for promoting precision treatment of gastric cancer.

### 3.2 Investigation of the carcinogenic mechanism of *Helicobacter pylori* using gastric organoid models

*Helicobacter pylori* (Hp) is a high-risk factor for gastric cancer, as its infection can trigger pathological changes in gastric epithelial tissues, ultimately leading to carcinogenesis (Amieva and Peek, 2016). Currently, research on this pathological process primarily relies on cell lines or animal models, both of which have significant limitations. Cell lines fail to replicate the complex tissue microenvironment *in vivo*, while animal models exhibit physiological differences due to species variation, making them unable to accurately simulate the interaction between *Helicobacter pylori* and human gastric tissues or the true pathological progression of gastric cancer. In contrast, gastric organoid models can closely recapitulate the physiological microenvironment of human gastric tissues, providing a more ideal research tool for in-depth analysis of the molecular mechanisms, cellular interactions, and pathological evolution involved in H. pylori-induced gastric cancer (Yu et al., 2025; Pompaiah and Bartfeld, 2017; Chakrabarti and Zavros, 2020). Wuputra et al. established gastric cancer organoid models and further constructed Hp-infected gastric cancer organoid models. They used cytotoxin-associated gene A (CagA)-green fluorescent protein (GFP)-labeled Hp to track the infection of gastric organoids and observe the oncogenic mechanism of Hp. The results revealed that hepatoma-derived growth factor (HDGF) and tumor necrosis factor α (TNFα) are independent signals involved in Hp-induced gastric cancer development (Wuputra et al., 2023). Liu and colleagues confirmed at the gastric organoid level that concurrent infection with Hp and Epstein-Barr virus (EBV) enhanced the proliferation of gastric organoids and altered their histological morphology (Liu et al., 2025). Schlaermann et al. isolated gastric gland cells from healthy human gastric sections or biopsies and established cell spheroid models. They transferred the organoid spheroids from the 3D culture environment to 2D standard culture dishes and constructed a primary cell model of Hp infection in the 2D planar model. Detection results showed that the infected primary cells exhibited all characteristics of successful infection and displayed responses similar to those of infected tissues. This model provides an excellent *in vitro* simulation tool for studies investigating the pathogenic mechanisms of Hp infection (Schlaermann et al., 2016). Hofer et al. established a homeostatic human gastric organoid-on-a-chip system that can model the structure of the human gastric epithelium and mimic the interactions between gastric epithelial cells under physiological and pathological conditions. This system is capable of sustaining long-term Hp infection and contributes to research on the disease progression induced by Hp (Hofer et al., 2025). Thus, gastric organoid models also play a certain role in exploring the carcinogenic mechanism of Hp.

### 3.3 Application of gastric cancer organoids in immunotherapy-related research

Immunotherapy has become the new standard for treating advanced or metastatic gastric cancer. In the clinical management of gastric cancer, immunotherapy is administered either as monotherapy or in combination with other treatment modalities. Significant progress has been achieved in current gastric cancer research on immunotherapy, which has greatly improved the survival rate of patients with advanced recurrent or metastatic gastric cancer (Högner and Moehler, 2022). Tumor immunotherapy mainly includes immune checkpoint inhibitors (ICIs), tumor-associated vaccines, and adoptive immunotherapy (Christodoulidis et al., 2024; Faghfuri et al., 2022). By blocking the interaction between immune checkpoint proteins, immune checkpoint inhibitors eliminate the immunosuppressive effect of immunosuppression-related molecules on the surface of tumor cells, thereby reactivating the ability of the body’s immune cells to kill tumors (Wang SJ. et al., 2023). Preventing tumorigenesis can largely reduce the number of cancer patients. Currently, the main approaches to prevent tumor development include avoiding exposure to carcinogens, administering vaccines against oncogenic viruses, and inoculating tumor-associated vaccines. Cancer vaccines primarily target tumor-associated antigens to trigger the body’s immune response against these specific antigens, thereby selectively targeting and eliminating cancer cells in the human body (Ruzzi et al., 2025). Currently, tumor vaccines can be mainly divided into two categories: preventive vaccines and therapeutic vaccines. Preventive vaccines primarily include HPV vaccines (Williamson, 2023) and hepatitis B vaccines (Cao et al., 2022), while therapeutic vaccines encompass viral vector vaccines (Wang S. et al., 2023), tumor antigen vaccines (Lang et al., 2022; Bhagat et al., 2023), nucleic acid vaccines (Kenoosh et al., 2024), and dendritic cell vaccines (Ding et al., 2021), among others. Adoptive immunotherapy involves the *in vitro* activation, genetic modification, or large-scale expansion of a patient’s own immune cells, which are then reinfused into the patient with enhanced anti-tumor activity. This approach aims to directly kill tumor cells and remodel the disordered tumor immune microenvironment, thereby achieving the goal of cancer treatment (Zhu et al., 2022; Albarrán et al., 2024). Adoptive immunotherapy mainly includes unmodified adoptive immunotherapies, such as tumor-infiltrating lymphocyte (TIL) therapy (Monberg et al., 2023), cytokine-induced killer (CIK) cell therapy (Sharma and Schmidt-Wolf, 2021), and natural killer (NK) cell therapy (Myers and Miller, 2021). Modified adoptive immunotherapies primarily include chimeric antigen receptor T-cell (CAR-T) therapy (Baker et al., 2023) and T-cell receptor-engineered T-cell (TCR-T) therapy (Baulu et al., 2023), among others. Although immunotherapy has significantly improved the prognosis of tumor patients, its efficacy in advanced gastric cancer remains limited. This is largely due to the complex tumor immune microenvironment composed of multiple components, as well as the diverse molecular subtypes and high heterogeneity of gastric cancer patients. As a result, immunotherapy only demonstrates certain efficacy in specific subtypes of gastric cancer (Yasuda and Wang, 2024). The process of tumorigenesis involves complex and continuous interactions between malignant tumor cells and the immune microenvironment. Immunotherapies have achieved favorable outcomes in clinical practice. However, there are significant differences in the response to immunotherapy across different tumor types. The development of experimental models that can well simulate the patient-specific immune microenvironment, recapitulate tumor biological characteristics, and reflect immunotherapeutic effects will enable the precision of immunotherapy. Organoids highly preserve the heterogeneity of tumor tissues, making them excellent models for immunotherapy research (Polak et al., 2024).

Patient-derived gastric cancer organoid-immune cell co-culture systems serve as ideal models for evaluating the *in vitro* efficacy of immunotherapy-related treatments. Ota et al. established gastric cancer organoid models and co-cultured them with peripheral blood mononuclear cells (PBMCs) and natural killer (NK) cells isolated from healthy individuals. Immune cells could move freely and make close contact with gastric cancer organoids, allowing the assessment of immune cell cytotoxicity. The results confirmed that co-culture with PBMCs killed more than 50% of gastric cancer cells, while co-culture with NK cells achieved over 70% cytotoxicity. This model enables *in vitro* efficacy testing of immune checkpoint inhibitors before administration to gastric cancer patients (Ota et al., 2023). Tumor cells expressing programmed death-ligand 1 (PD-L1) can specifically bind to programmed death-1 (PD-1) on the surface of CD8^+^ cytotoxic T lymphocytes (CTLs), initiating immunosuppressive signals to evade immune surveillance and destruction. In gastric cancer, approximately 40% of tumor tissues express PD-L1, but the response rate to immunotherapy in these patients is only 30%, making research on personalized immunotherapy for gastric cancer crucial. Chakrabarti and colleagues established a co-culture system centered on patient-derived gastric cancer organoids combined with immune cells. This system can simulate the dynamic interactions between immune cells and tumor cells in the *in vivo* tumor microenvironment, providing an ideal preclinical model for deciphering immune regulatory mechanisms and screening personalized immunotherapy regimens (Chakrabarti et al., 2021). Notably, tumor cells expressing PD-L1 evade immune surveillance and destruction by binding to PD-1 on CD8^+^ CTLs, triggering immunosuppressive signals. In gastric cancer, around 40% of tumor tissues express PD-L1, but only approximately 30% of these patients respond effectively to immunotherapy. This highlights the importance of personalized immunotherapy research for gastric cancer. Chakrabarti et al. constructed a co-culture system based on patient-derived gastric cancer organoids and immune cells. This system recapitulates the dynamic crosstalk between immune cells and tumor cells in the tumor microenvironment, offering an optimal preclinical model for exploring immune regulatory mechanisms and selecting personalized immunotherapeutic strategies (Chakrabarti et al., 2021).

In summary, gastric cancer organoid-immune cell co-culture models can highly mimic the interactions between tumor cells and immune cells in the *in vivo* tumor microenvironment. They not only reproduce key processes such as signal transduction, cell migration, and cytotoxicity but also retain the heterogeneity and immune characteristics of patients’ tumors, providing high-quality personalized preclinical models for gastric cancer immunotherapy research. The establishment of this model not only facilitates a better understanding of gastric cancer immune escape mechanisms and the screening of potential immunotherapeutic targets but also optimizes immunotherapy regimens and predicts patient responses to immunotherapy, further advancing the translation of gastric cancer immunotherapy from basic research to clinical practice.

### 3.4 Application of gastric cancer organoids in exploring the molecular mechanisms of gastric cancer progression and chemotherapy resistance

Gastric organoids, as an *in vitro* experimental model, offer advantages over animal models and conventional cell models in multiple aspects. Particularly, human gastric tissue-derived gastric organoids retain patient-specific tissue characteristics, enabling the study of factors influencing gastric cancer progression *ex vivo*. As a novel preclinical model for gastric cancer, organoids have also played a significant role in research on the molecular mechanisms underlying gastric cancer progression and drug resistance (Fan et al., 2018). CRISPR/Cas9 technology has been widely used to explore the functions of cancer-related genes, establish tumor-bearing animal models, and identify drug targets (Wang et al., 2022). However, the application of CRISPR/Cas9 in gastric cancer organoids is relatively limited. Mircetic and colleagues employ CRISPR/Cas9 in gastric cancer organoids, demonstrating that KDM1A is crucial for the survival of gastric cancer cells. They found that inhibiting KDM1A impaired the growth of patient-derived gastric cancer organoids, suggesting that KDM1A inhibitors could be potential therapeutic agents for gastric cancer patients (Mircetic et al., 2023). Lo et al. used CRISPR/Cas9 in wild-type human gastric organoids to construct an ARID1A deficient oncogenic transformation model, revealing that ARID1A loss induces gastric mucinous metaplasia by suppressing Wnt/β-catenin activity (Lo et al., 2021). Chemotherapy resistance is a major cause of treatment failure in gastric cancer, and identifying resistance-related targets is currently a key focus in addressing this issue (Zhang et al., 2022). While numerous studies on gastric cancer drug resistance have been conducted at the conventional cell line level, most findings fail to translate into clinical applications, likely because cell lines do not adequately reflect individual patient responses to therapy. Gastric cancer organoid models effectively address this limitation. Yang et al. established docetaxel-sensitive and docetaxel-resistant gastric cancer organoids. Single-cell RNA sequencing (scRNA-seq) revealed significant changes in the cell populations between sensitive and resistant organoids, and identified that drug resistance-related genes FOS, IFI27, and PTTG1IP were upregulated in both resistant organoids and gastric cancer patients. Knockdown of FOS, IFI27, and PTTG1IP enhanced docetaxel sensitivity in both cell lines and organoids, and regulated the production of reactive oxygen species (ROS), autophagy, and apoptosis. Pseudotime trajectory analysis indicated that resistant cells mainly occupied the terminal differentiation stage. This study confirmed that FOS, IFI27, and PTTG1IP may serve as potential therapeutic targets for patients with docetaxel-resistant gastric cancer (Yang et al., 2025). Wong et al. found that the interferon/JAK/STAT signaling pathway was activated in 5-FU and cisplatin-resistant gastric cancer organoids, inducing the expression of ADAR1. To clarify whether ADAR1 is associated with gastric cancer cell resistance to 5-FU and cisplatin, ADAR1 was subjected to mutation and knockdown in gastric cancer organoid models. The results showed that both wild-type and ADAR1-knockdown groups exhibited enhanced cell proliferation and increased sensitivity to chemotherapeutic drugs. Thus, ADAR1 overexpression is considered to be associated with the development of chemoresistance in gastric cancer patients. Further studies revealed that ADAR1 mediates gastric cancer chemoresistance through editing SCD1 RNA (Wong et al., 2023). Currently, most studies investigating the molecular mechanisms of gastric cancer progression and therapeutic tolerance using organoid models are conducted in conjunction with cell experiments. Based on its two major advantages—preserving the specificity of gastric cancer patients and better simulating the *in vivo* tumor microenvironment—the gastric cancer organoid model has become an ideal tool for exploring the key mechanisms of gastric cancer progression and the molecular basis of chemoresistance, providing strong support for in-depth research into gastric cancer pathological mechanisms. Relevant studies on the molecular mechanisms of gastric cancer progression based on gastric cancer organoid models are summarized in Table 1.

**TABLE 1:** Investigation of molecular mechanisms underlying gastric cancer progression and therapeutic resistance based on gastric cancer organoid models.

Experimental models	Year	Country	Research content and results	Authors and references
Cell lines + Tumor spheroids + Organoids	2024	China	This study confirmed low BATF2 expression in gastric cancer epithelial cells via single-cell RNA sequencing. Survival analysis revealed that patients with high BATF2 expression had a favorable prognosis, and high BATF2 expression significantly reduced cancer stem cell markers in gastric cancer cells. Further research verified that BATF2 enhanced the sensitivity of gastric cancer cells to 5-Fu by inhibiting the PTEN/AKT/β-catenin/ABCG2 signaling pathway	Cao L, Weng K, Li L, et al. (Cao et al., 2024)
Cell lines + Organoids	2023	China	This study demonstrated that VPS35 is highly expressed in tumor tissues, and VPS35 promotes gastric cancer cell proliferation by upregulating CDK2 and cyclin D. Further investigation found that VPS35 can inhibit the degradation of EGFR, resulting in the downstream activation of the ERK1/2 pathway. Meanwhile, the study revealed that VPS35 increases the sensitivity of gastric cancer to EGFR inhibitors	Yu J, Feng H, Sang Q, et al. (Yu et al., 2023)
Cell lines + Organoids	2024	China	The results of this study showed that NUF2 is highly expressed in gastric cancer tissues, and gastric cancer patients with high NUF2 expression have poor overall survival (OS). High NUF2 expression can activate the MAPK pathway to promote gastric cancer cell proliferation. Quercetin, a NUF2 inhibitor, can inhibit tumor growth in organoid and PDX models	Long B, Zhou H, Xiao L, et al. (Long et al., 2024)
Cell lines + Organoids	2025	China	ASF1B is highly expressed in gastric cancer tissues and associated with poor prognosis in patients. ASF1B activates the PI3K/AKT and ERK1/2 signaling pathways by inhibiting the expression of H2AC20, thereby promoting tumor cell proliferation, colony formation, invasion, and migration	Zhao M, Zhang J, He Y, You C. (Zhao et al., 2025)
Cell lines + Organoids	2023	China	ATOH1 is lowly expressed in gastric cancer tissues. ATOH1 targets and regulates the transcription of GAS1 to activate the AKT/mTOR signaling pathway, thereby driving the stemness and progression of gastric adenocarcinoma	Zhong Q, Wang HG, Yang JH, et al. (Zhong et al., 2023)
Cell lines + Organoids	2022	China	EXOSC5 is highly expressed in gastric cancer tissues and cell lines, and its overexpression promotes the proliferation of gastric cancer cells and organoids. Mechanistic studies revealed that EXOSC5 promotes the expression of cyclin D1 and inhibits the expression of p21 and p27 by regulating the AKT/STAT3 pathway, thereby facilitating gastric cancer proliferation	Chen X, Huang Y, Liu J, et al. (Chen et al., 2022)

### 3.5 Practical application of gastric cancer organoids in personalized treatment of gastric cancer

As mentioned earlier, gastric cancer organoids have demonstrated significant advantages in various areas, including drug sensitivity testing for patients, basic research on the mechanisms of gastric cancer progression, and the development of immunotherapies for gastric cancer. In practical applications, patient-derived organoid-based drug sensitivity testing has emerged as a promising ideal model and a key technological approach for achieving personalized treatment in gastric cancer patients. Multiple studies have confirmed that the drug sensitivity of gastric cancer organoids is highly consistent with the actual drug sensitivity responses observed in patients. In clinical practice, patient-derived gastric cancer organoid models can be constructed to perform high-throughput screening of chemotherapy, targeted therapy, and immunotherapy drugs. This enables rapid identification of the most effective drugs for individual patients and helps determine the treatment regimen likely to yield the best prognosis. This approach effectively avoids the adverse effects and resource wastage associated with ineffective treatments resulting from traditional empirical medication. Moreover, organoid models have been utilized in clinical trials related to gastric cancer. For example, in a randomized phase III trial by Smyth et al. (2021) titled “EGFR amplification and outcome in a randomized phase III trial of chemotherapy alone or chemotherapy plus panitumumab for advanced gastro-oesophageal cancers,” the lack of commercially available EGFR-amplified gastroesophageal adenocarcinoma (GEA) cell lines hindered preclinical testing of EGFR inhibitors (EGFRi) in gastric cancer. To address this, the research team developed PDO models from patients with EGFR-amplified metastatic gastroesophageal cancer to conduct preclinical experiments and evaluate patient responses to treatment. Furthermore, constructing patient-derived organoid models at different disease stages allows for comparative analysis of changes in drug sensitivity over time and facilitates the exploration of potential mutation targets and mechanisms related to drug resistance. Particularly for patients with locally advanced or rare pathological types of gastric cancer, for whom effective treatment options are currently limited, organoid models offer a potential therapeutic aid and are expected to advance the progress of personalized treatment.

## 4 Challenges and prospects

Currently, organoid technology has been widely applied in tumor-related research, including the establishment of tumor models and biobanks, the development of antitumor drugs, tumor immunotherapy, personalized therapy, and the study of tumor progression (Lin et al., 2022). Although gastric cancer organoids offer the advantage of preserving the heterogeneity of tumor tissues, they also have certain limitations that hinder their clinical application. For instance, tumors exhibit extensive subtypes, and the *in vitro* expansion efficiency of organoids is unpredictable. Additionally, conventional organoid cultures typically contain only tumor cells, lacking other cell types and nutritional components of the immune microenvironment. This deficiency prevents them from fully simulating the *in vivo* survival environment of tumor tissues (LeSavage et al., 2022). While results from some current studies indicate a high consistency between the drug response of organoids and that of patients, these findings are based on small-sample clinical data and lack further validation with large cohorts (Veninga and Voest, 2021). A significant limitation of the organoid system is its lack of inter-organ communication capability. Currently available patient-derived organoid systems essentially only simulate the characteristics of local human tissues and cannot recapitulate the physiological state of the entire organism. However, the human body is an organic whole where systems such as the circulatory, immune, digestive, and endocrine systems are closely interconnected. These systems regulate each other through mechanisms like signaling molecule transmission and metabolite exchange, and no tissue or organ can function independently of the overall bodily environment. The absence of such systemic connections makes it difficult for existing organoids to fully simulate the complex pathophysiological processes *in vivo*. Therefore, further optimization of organoid technology is required to enhance their structural and functional similarity to the physiological state of the human body, thereby improving the clinical translational value of these models (Thorel et al., 2024; Kim et al., 2020). In recent years, the combination of organoids with microfluidic systems to form organ-on-a-chip technology has largely overcome these shortcomings, and this technology is continuously being improved according to research needs. Additionally, as there are currently no unified standards for the establishment and culture of organoids, the success rate and functional consistency of organoid cultures vary significantly, leading to poor reproducibility of related experiments. Furthermore, compared to conventional cell line cultures, organoid culture not only requires expensive Matrigel but also needs various cytokines in the culture medium, making it more costly.

Although organoid technology has demonstrated indispensable and significant value in personalized treatment for patients, particularly in the field of clinical drug sensitivity testing, where it facilitates the formulation of precision treatment plans tailored to each patient and provides critical support for optimizing clinical outcomes, the clinical translation and large-scale application of this technology still face numerous formidable challenges (Peng et al., 2025). Although organoids retain the specificity of tumor tissue, they cannot accurately mimic the in vivosurvival environment of tumors. Organ-on-a-chip technology, which combines organoids with microfluidic chips, can compensate for this defect. Organ-on-a-chip is a cutting-edge technology platform that primarily uses engineering methods to construct a miniature system on a chip containing microchannels and culture chambers. This allows for precise control over the delivery, circulation, and mechanical stimulation of fluids like culture medium and drugs, thereby providing a highly biomimetic, dynamic, and controllable microenvironment for the 3D-cultured organoids. Its main advantage lies in its ability to simulate the complex in vivomicroenvironment. Organ-on-a-chip can simulate fluid flow and mechanical forces generated by tissue compression and gases in the blood, making the entire cellular environment closer to that of a real organ (Cauli et al., 2023; Zarrintaj et al., 2022).3D printing technology is now widely used in various fields. It primarily involves obtaining a three-dimensional digital model of an object through 3D scanning or computer-aided design, and then building complex tissue structures by gradually layering materials (Hossain et al., 2021). Currently, 3D printing is extensively applied in healthcare, such as in heart tissue engineering (Wang et al., 2021), bone substitutes for orthopedic surgery (Ansari et al., 2022), and dentures in dentistry (Alzahrani et al., 2023), among others. The innovative integration of 3D printing technology with organoids can significantly enhance the simulation of human physiological and pathological states by precisely constructing the tumor microenvironment. The convergence of these two technologies provides an experimental model for in vitrocancer research that is closer to the in vivoscenario, enabling more accurate reproduction of tumor cell proliferation, invasion, and interaction with the immune microenvironment. This, in turn, accelerates the efficiency of anti-cancer drug screening and the process of clinical translation (Luo et al., 2021). Furthermore, artificial intelligence (AI) and machine learning hold distinct advantages in predicting the phenotypes and drug responses of organoids. AI technology is capable of translating the biological characteristics of gastric cancer organoids into computational models, providing a reliable tool for data analysis and prediction. Simultaneously, AI algorithms can guide the differentiation of organoids to generate more precise models tailored to specific research needs (Meng et al., 2025; Wang et al., 2025). Although patient-derived tumor organoids, as a research model, still have many shortcomings, integrating organoids with advanced technologies such as organ-on-a-chip, 3D bioprinting, CRISPR-Cas9 gene editing, and co-culture with immune cells will further promote their translation into clinical applications (Qu et al., 2021).

Currently, organoid models are expanding their application dimensions and scientific value through interdisciplinary integration with cutting-edge technologies such as genomics, bioengineering, and artificial intelligence. This has become a significant development trend and a notable advantage in the field of organoid research. In this article, we systematically review the research progress of gastric cancer organoids in fundamental mechanism studies, drug sensitivity evaluation, and immunotherapy development, and provide a comprehensive summary of their clinical translation potential. However, compared to other articles on the research and application of organoids, this paper does not delve deeply into the specific mechanisms and synergistic advantages of integrating organoids with emerging technologies such as microfluidic chips, live dynamic imaging, and spatial multi-omics analysis. Nor does it thoroughly explore the detailed applications of organoids in broader scenarios, such as constructing dynamic disease progression models, high-throughput drug screening platforms, and precision companion diagnostic systems. In recent years, numerous reviews have extensively discussed the practical pathways of integrating organoids with other technologies from various perspectives. For example, in the integration of organoids with microfluidic technology, multiple studies have successfully developed “organ-on-a-chip” systems. These systems can more realistically simulate the hydrodynamic characteristics and cell-cell interactions within the tumor microenvironment, thereby demonstrating unique value in assessing drug permeability and toxicity. Additionally, by combining organoid models with single-cell sequencing and spatial transcriptomics, researchers can gain deeper insights into the evolutionary patterns of tumor heterogeneity during treatment, offering a dynamic perspective for studying drug resistance mechanisms. Furthermore, when integrated with artificial intelligence-based image analysis, the phenotypic recognition accuracy of organoids has been significantly enhanced, providing new possibilities for large-scale drug discovery and biomarker identification. We believe that with the standardization of organoid culture systems, the maturation of technology integration pathways, and the expansion of clinical validation, organoid models will undoubtedly play a critical role in the future precision oncology ecosystem. They can not only serve as reliable surrogates for patient drug sensitivity testing but also hold the potential to become comprehensive tools spanning drug development, treatment optimization, and dynamic efficacy monitoring. Ultimately, they will contribute to the widespread clinical implementation of personalized treatment for gastric cancer and other cancer types. Future research should place greater emphasis on the systematic evaluation of interdisciplinary technology integration and strengthen the validation and application of organoids in real-world clinical scenarios.
